# Long-term trends and projections of CT and MRI utilization across selected OECD countries: a framework for sustainable imaging service planning

**DOI:** 10.1186/s13244-026-02458-3

**Published:** 2026-11-05

**Authors:** Giovanni Aguzzi, Katia Iaccarino, Luca Di Palma, Isabella Castiglioni, Amelia Compagni, Marco Alì

**Affiliations:** 1 CDI Centro Diagnostico Italiano S.p.A., Milan, Italy; 2 Department of Physics, University of Milano–Bicocca, Milan, Italy; 3 Department of Social and Political Sciences, Bocconi University, Milan, Italy

**Keywords:** Magnetic resonance imaging, Computed tomography, Demographic ageing, Scanner productivity, Health system sustainability

## Abstract

**Objectives:**

CT and MRI utilization is increasing across high-income countries, partly because of population ageing. This study integrated cross-country trends in examination rates, demographic change, and scanner productivity across OECD countries to project utilization to 2050 and inform sustainable service planning.

**Materials and methods:**

CT and MRI examination rates were obtained from the OECD Data Explorer and Japan’s National Database of Health Insurance Claims Open Data. Population data were derived from the United Nations World Population Prospects 2024. Linear regression projected examination rates to 2050. Pearson coefficients assessed associations between imaging rates and population shares aged 0–14, 15–64, and ≥ 65 years. Scanner productivity was calculated as annual examinations per device.

**Results:**

CT utilization was strongly associated with the population aged ≥ 65 years in all countries (r = 0.95–1.00). MRI showed similarly strong associations in most countries (r = 0.95–0.99), with Italy as the main exception. Linear extrapolation projected CT rates of approximately 722 examinations per 1000 population in Korea and 520 in the United States by 2050, while MRI rates reached approximately 327 in France and 308 in Germany. Population-level utilization did not consistently correspond to device-level productivity; CT examinations per scanner ranged from 2153.3 in Japan to 11,526.5 in Canada.

**Conclusions:**

Population ageing was associated with increasing CT and MRI utilization. Future demand may also depend on health system organization and scanner productivity. Sustainable planning should integrate demographic forecasting, appropriateness stewardship, operational optimization, workforce development, and technological innovation rather than rely solely on proportional capacity expansion.

**Key Points:**

***Question*** Ageing drives CT/MRI demand (r = 0.95–1.00 for CT); projected to continue to 2050, offering a valuable planning window.

***Findings*** High utilization ≠ high scanner productivity; operational organization shapes imaging output independently of demand volume.

***Critical relevance statement*** Sustainable planning requires demographic forecasting, appropriateness management, and technological innovation, not just capacity expansion.

**Graphical Abstract:**

**Figure:**
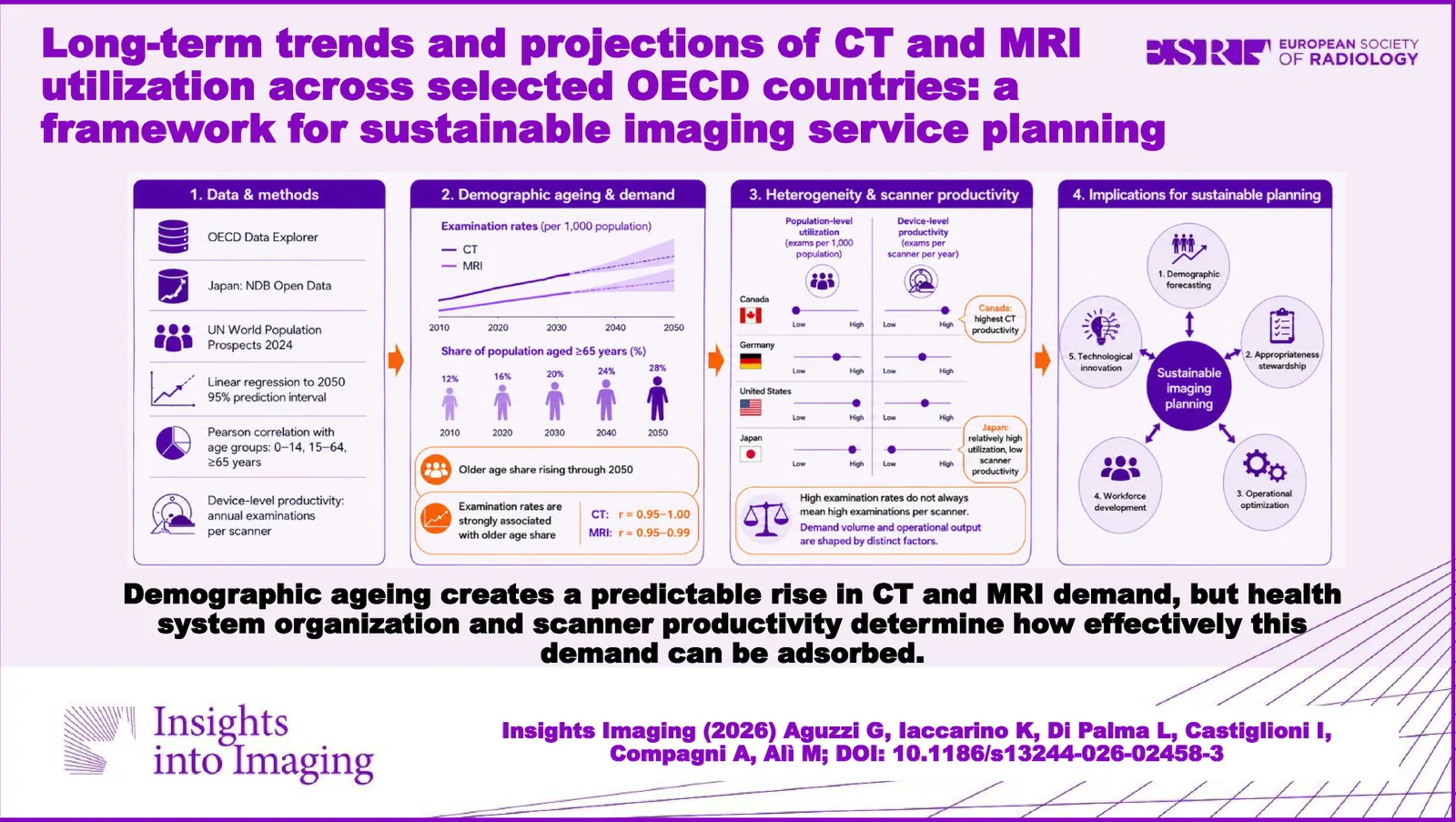

## Introduction

Advanced diagnostic imaging occupies a central role in modern radiology, underpinning diagnostic accuracy, disease staging, and therapeutic decision-making across a wide range of pathways [1–3]. Among imaging modalities, magnetic resonance imaging (MRI) and computed tomography (CT) are distinguished by high clinical impact but also by considerable requirements in terms of capital investment, specialized workforce, and operational costs [4, 5].

Over recent decades, both modalities have diffused widely across high-income countries, yet this expansion has been markedly heterogeneous. International comparisons consistently reveal pronounced cross-country variation in scanner density and in examination volumes per device, even among countries with broadly comparable population sizes, income levels, and healthcare expenditure [6, 7]. Such variability suggests that factors beyond population scale, including organizational models, referral practices, and reimbursement mechanisms, play a substantial role in shaping observed imaging workloads [8, 9].

Alongside these structural differences, population ageing has emerged as a major determinant of imaging demand. Population-based evidence has shown that CT and MRI utilization increases substantially with age. In the study by Smith-Bindman et al, conducted across US healthcare systems in 2016, age-specific utilization rates increased substantially from children to older adults for both modalities (Table 1) [10].

**Table 1:** Age-specific CT and MRI utilization rates in US healthcare systems in 2016

Modality	Children	Adults aged 18–64 years	Adults aged ≥ 65 years
CT	22	134	428
MRI	21	85	139

Values are expressed as examinations per 1000 person-years. Source: Smith-Bindman et al [10]

As the share of adults aged 65 years and older continues to rise across OECD countries, projected on average from 18.5% in 2023 to 26.4% by 2050 [11], demographic change represents a structural and largely predictable factor in future imaging demand rather than a transient phenomenon.

These dynamics carry important implications for healthcare sustainability. Capacity expansion, the most common response to increasing demand, requires substantial capital investment and may generate supply-induced utilization without addressing underlying inefficiencies [12, 13]. Evidence from Canada further suggests that scanner availability alone does not determine imaging output: despite having fewer CT and MRI scanners per million population than the OECD average, Canada achieved substantially higher numbers of examinations per scanner, highlighting the role of throughput and operational organization in shaping imaging capacity [14]. At the same time, increasing imaging volumes must be considered in relation to radiology workforce capacity, as recent projections indicate that future radiologist supply is strongly influenced by training capacity and workforce attrition [15]. In the context of increasing fiscal pressures and workforce constraints, meeting future imaging demand will likely require a combination of capacity planning and efficiency-oriented strategies. Technological advances, workflow optimization, optimized scheduling, and better use of existing scanner and workforce capacity may help sustain access and quality in MRI and CT services while limiting the need for proportional infrastructure expansion [16–18].

Against this background, this study pursues three objectives: (1) to describe cross-country patterns and long-term trends in CT and MRI examination rates across selected OECD countries; (2) to project CT and MRI examination rates to 2050 and examine their association with changes in population age structure; and (3) to discuss efficiency-oriented strategies that may support the long-term sustainability of advanced diagnostic imaging across its economic, environmental, and social dimensions.

## Materials and methods

### Study design

This study was designed as a descriptive and projection-based analysis of CT and MRI examination rates across selected OECD countries. No patient-level data were used. All analyses were performed using aggregated, publicly available data. Countries were selected using a maximum-variation purposive sampling strategy rather than with the aim of providing exhaustive OECD coverage. The analytical sample was designed to capture heterogeneity in geographical setting, demographic ageing trajectories, healthcare-system organization, and baseline CT and MRI utilization, while requiring sufficiently long and reasonably continuous annual time series for both modalities to support longitudinal trend estimation. The included countries should therefore be interpreted as comparative case studies and not as a statistically representative sample of all OECD members. Japan was retained as a secondary extreme-case comparator because it represents an advanced ageing setting, combining the highest baseline share of adults aged ≥ 65 years among the countries examined with high utilization of advanced diagnostic imaging. This made Japan particularly informative for exploring imaging demand in a mature ageing population, although its longitudinal utilization data were obtained from the national NDB Open Data source rather than from the OECD series. Ethics approval was not required because the study exclusively used publicly available, aggregated, and non-identifiable data, with no access to individual-level health information.

### Imaging utilization data

CT and MRI examination rates were obtained from the OECD Data Explorer [19], within the “Health” topic, “Health care use” section, and “Diagnostic exams” dataset. Data were extracted as annual values and expressed as the number of examinations per 1000 population. For each country, the observed period was defined according to the first year in which both CT and MRI examination data were available in the OECD dataset. Observed OECD data were used up to the most recent available year, and subsequent values were projected to 2050.

For Japan, the OECD diagnostic examination dataset contained only a single available observation for CT and MRI examination rates in 2014, which was insufficient for time-series analysis and projection. Therefore, Japanese CT and MRI examination counts were extracted separately from the National Database of Health Insurance Claims and Specific Health Checkups of Japan (NDB) Open Data [20]. Annual examination rates were calculated by dividing the number of CT or MRI examinations by the corresponding national population and were expressed per 1000 population.

### Population data

Population data were obtained from the United Nations World Population Prospects 2024 [21]. For each country, annual population values were extracted for three age groups: 0–14 years, 15–64 years, and 65 years and older, normalized and expressed per 1000 population. The demographic period was aligned with the corresponding imaging utilization period, extending from the first year with available CT and MRI data to 2050. Historical population estimates were used for observed years, whereas population projections were used for the period extending to 2050.

### Projection of CT and MRI examination rates

Future CT and MRI examination rates were projected up to 2050 using a linear regression model fitted to the observed annual examination rates, with calendar year as the explanatory variable and examination rate per 1000 population as the outcome. The goodness of fit of each country- and modality-specific model was assessed using the coefficient of determination (R²), which was calculated exclusively from the observed historical examination rates; projected values were not included. For each projected value, a 95% prediction interval (PI) was calculated. The projections were interpreted as linear extrapolations based on historical utilization trends and not as forecasts able to account for future changes in clinical guidelines, imaging technologies, healthcare capacity, reimbursement policies, population health status, or access to diagnostic services.

### Correlation analysis

Pearson correlation coefficients were calculated between observed annual CT and MRI examination rates per 1000 population and the normalized population shares of the three age groups, for each country and modality over the full analytical period. All correlations were interpreted as measures of association and not as evidence of causality.

### Scanner productivity

Examinations per scanner (the annual number of CT or MRI examinations performed per installed scanner) were extracted from the OECD dataset for descriptive comparison, using the most recent available year per country. For Japan, only a 2014 OECD value was available and was retained with caution.

### Data visualization

Country-specific trends were visualized separately for CT and MRI, plotting observed rates, projected rates, 95% PI, and age-group population shares against calendar year. Examination rates and population shares for the 0–14 and ≥ 65 age groups were displayed on the primary *y*-axis; the 15–64 age group on the secondary *y*-axis, given its larger absolute values.

## Results

### Demographic ageing across country-specific analytical periods

All included countries showed a progressive increase in the share of the population aged 65 years and older over the analytical period (Table 2).

**Table 2:** Population aged 65 years and older in selected OECD countries, from country-specific baseline year to 2050

Country	Analytical period	Start year	65+ at start (%)	2019 (%)	2023 (%)	2030 (%)	2050 (%)
Australia	2007–2050	2007	13.1	15.9	17.4	19.8	23.9
Canada	2003–2050	2003	12.8	17.5	19.4	22.5	25.7
France	2006–2050	2006	16.4	20.4	21.7	24.4	27.6
Germany	2005–2050	2005	18.9	21.6	22.8	26.5	30.3
Italy	2013–2050	2013	21.3	23.1	24.2	28	36.8
Japan	2014–2050	2014	26.2	28.6	29.6	31.1	37.5
Korea	2007–2050	2007	9.9	15.1	18.3	25.1	39.7
Spain	2010–2050	2010	16.9	19.4	20.7	24.6	36.1
USA	1995–2050	1995	12.4	15.7	17.4	20.4	23.1

Values are expressed as percentages of the total national population. The baseline year corresponds to the first year with available CT and MRI examination data. Source: authors’ elaboration based on United Nations World Population Prospects 2024 [20, 21]

By 2050, Korea showed the largest projected increase (from 9.9% in 2007 to 39.7% in 2050), followed by Spain (from 16.9% to 36.1%) and Italy (21.3% to 36.8%). Japan started from the highest baseline and remained among the oldest countries, rising from 26.2% in 2014 to 37.5%. More moderate increases were projected for Australia, Canada, France, Germany, and the United States. Despite differences in baseline age structure and pace of change, the direction of demographic change was consistent across all countries.

### Observed and projected CT and MRI examination rates

**Fig. 1:**
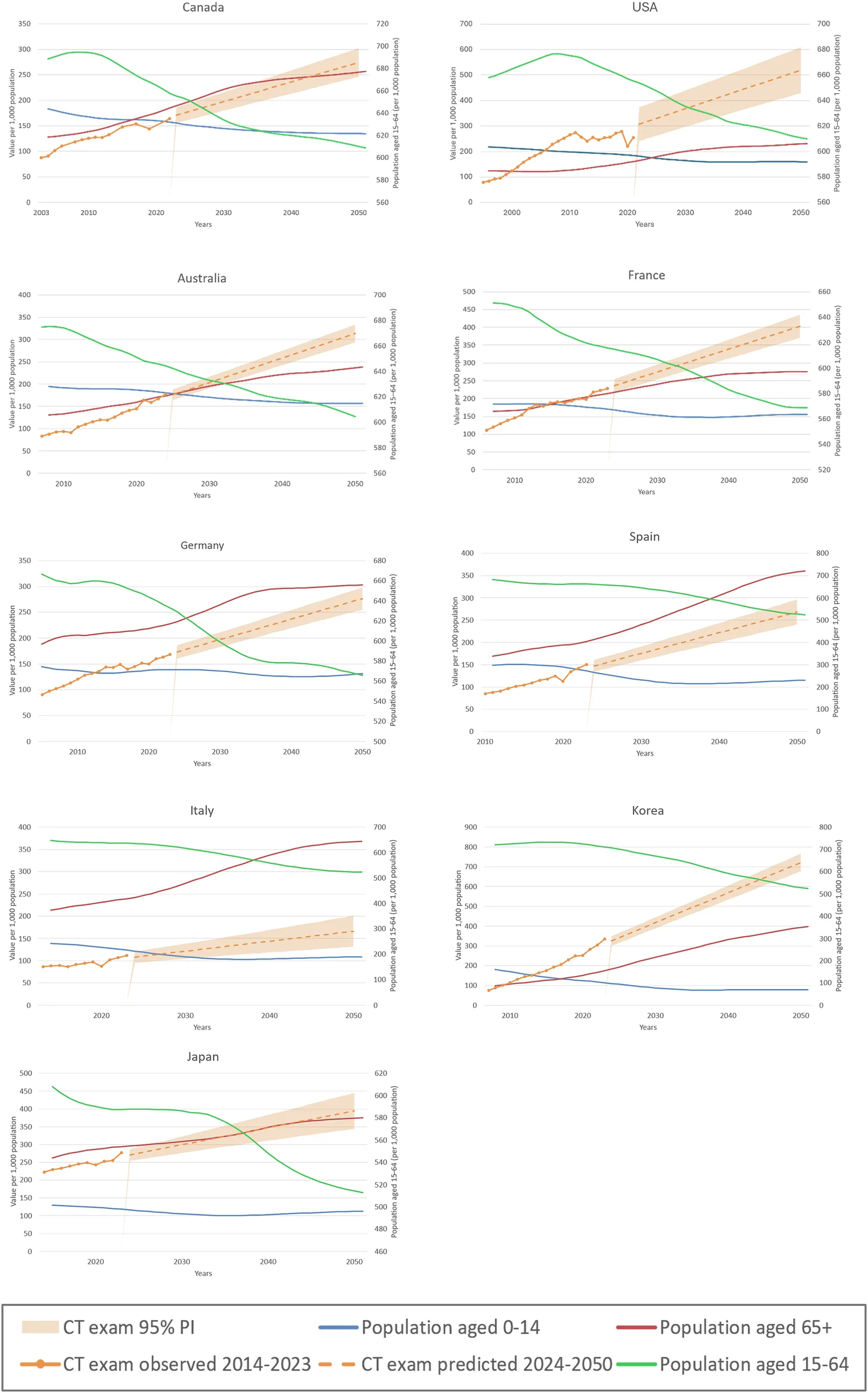
Country-specific CT examination trends, projections to 2050, and population age structure. Observed examination rates are shown as orange solid lines with markers, projected values as orange dashed lines, and 95% PI as shaded orange bands. Examination rates and population age groups 0–14 and ≥ 65 years are displayed on the primary *y*-axis, while the population aged 15–64 years is displayed on the secondary *y*-axis. All values are expressed per 1000 population

**Fig. 2:**
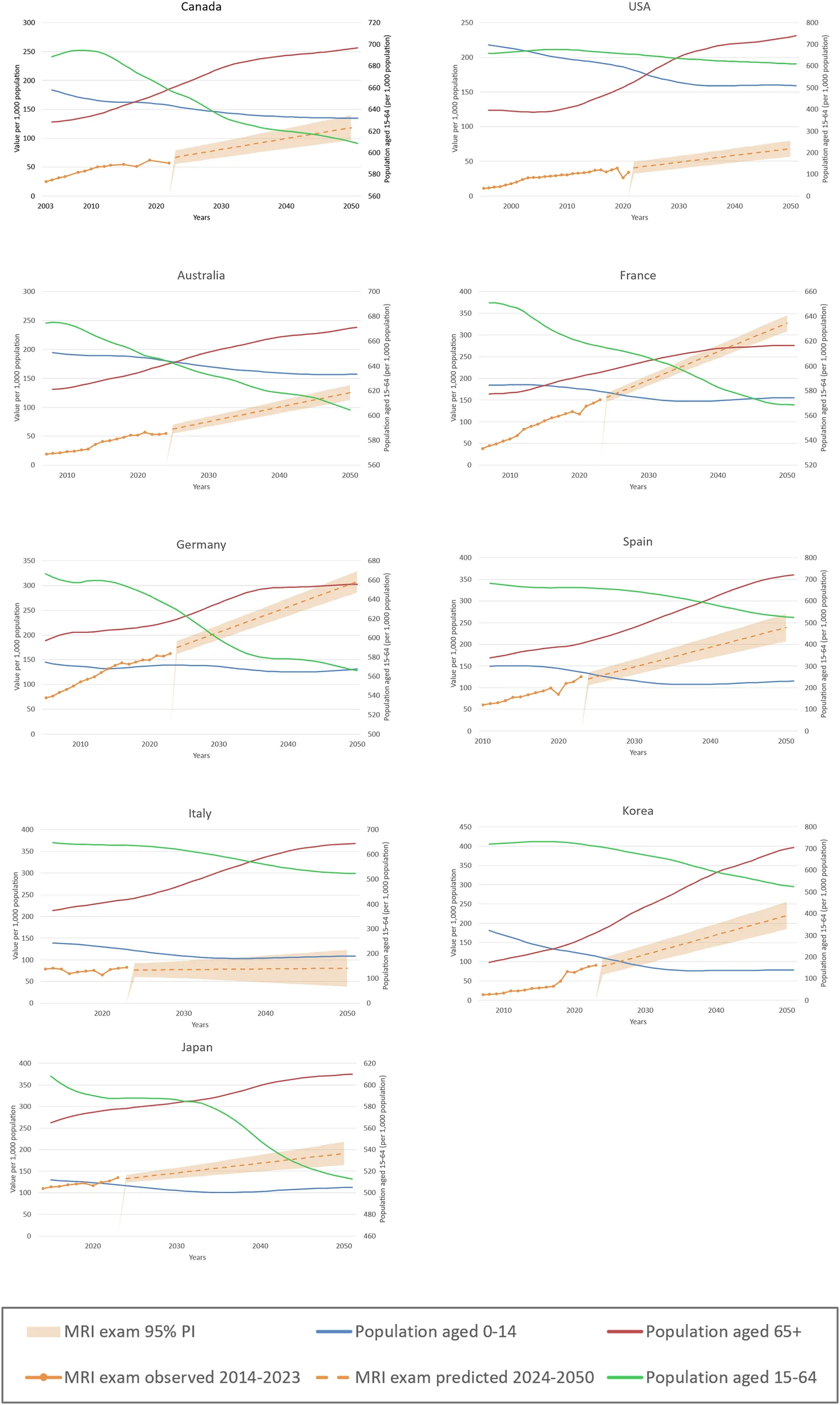
Country-specific MRI examination trends, projections to 2050, and population age structure. Observed examination rates are shown as orange solid lines with markers, projected values as orange dashed lines, and 95% PI as shaded orange bands. Examination rates and population age groups 0–14 and ≥ 65 years are displayed on the primary *y*-axis, while the population aged 15–64 years is displayed on the secondary *y*-axis. All values are expressed per 1000 population

Country-specific CT and MRI examination rates showed marked heterogeneity in both observed utilization levels and projected trajectories (Figs. 1 and 2). For CT, Korea showed the highest latest observed examination rate (333.5 examinations per 1000 population in 2023), followed by Japan (277.3), and the United States (254.5); Italy showed the lowest rate (111.1). Linear projections indicated continued increases in all countries, with Korea reaching approximately 722 examinations per 1000 population by 2050, the United States approximately 520, and Italy approximately 166. For MRI, Germany and France showed the highest observed rates in 2023 (161.9 and 150.8, respectively), while Canada and Australia showed the lowest (56.9 and 54.8). By 2050, France and Germany were projected to maintain the highest MRI rates (approximately 327 and 308, respectively), while Italy displayed a comparatively flat trajectory, remaining around 81 examinations per 1000 population. Across both modalities, 95% PI widened progressively over time, reflecting increasing uncertainty in longer-range projections. The goodness of fit of the country- and modality-specific linear regression models is reported in Table 3. For CT, R² values ranged from 0.73 in Italy to 0.98 in Korea, indicating a high goodness-of-fit. For MRI, R² values ranged from 0.01 in Italy to 0.99 in France. Italy represented the main exception, as its MRI examination rates fluctuated around a relatively stable level and did not show a consistent linear temporal trend. Germany MRI showed a high goodness-of-fit (R² = 0.97), despite the apparent flattening in the most recent observations, whereas the USA CT model showed a lower but still high goodness-of-fit (R² = 0.81).

**Table 3:** Goodness of fit of country- and modality-specific linear regression models fitted to observed CT and MRI examination rates

Country	Period	CT R²	MRI R²
Australia	2007–2024	0.97	0.95
Canada	2003–2022	0.94	0.86
France	2006–2023	0.95	0.99
Germany	2005–2023	0.95	0.97
Italy	2013–2023	0.73	0.01
Japan	2014–2023	0.87	0.84
Korea	2007–2023	0.98	0.89
Spain	2010–2023	0.94	0.92
USA	1995–2021	0.81	0.81

R² represents the coefficient of determination and indicates the proportion of variability in the observed examination rates explained by the linear temporal trend. Only historical observed values were included [19, 20]

### Correlation between imaging utilization and population age structure

CT examination rates were strongly and positively correlated with the population aged 65 years and older in all countries, with Pearson coefficients ranging from 0.95 (United States) to 1.00 (Korea), and negatively correlated with the 15–64 and 0–14 age groups (Table 4). A similar pattern was observed for MRI in most countries, with correlation coefficients ranging from 0.95 to 0.99. Italy represented the main exception, showing weaker associations across all age groups for MRI. These correlations should be interpreted as descriptive associations rather than evidence of causality, as imaging utilization may also be influenced by clinical practice, technology diffusion, healthcare capacity, reimbursement policies, and access to diagnostic services.

**Table 4:** Correlation between CT and MRI examination rates and population age structure across the full analytical period

Country	Period	Imaging modality					
		CT			MRI		
		65+	15–64	0–14	65+	15–64	0–14
Australia	2007–2050	0.99	−0.99	−0.98	0.99	−0.99	−0.97
Canada	2003–2050	0.98	−0.97	−0.97	0.97	−0.96	−0.97
France	2006–2050	0.98	−0.99	−0.87	0.98	−0.99	−0.87
Germany	2005–2050	0.97	−0.97	−0.78	0.97	−0.97	−0.78
Italy	2013–2050	0.99	−0.97	−0.83	0.51	−0.50	−0.45
Japan	2014–2050	0.99	−0.94	−0.65	0.99	−0.94	−0.65
Korea	2007–2050	1	−0.97	−0.92	0.99	−0.97	−0.91
Spain	2010–2050	0.99	−0.95	−0.88	0.99	−0.95	−0.88
USA	1995–2050	0.95	−0.89	−0.95	0.95	−0.90	−0.95

Values represent Pearson correlation coefficients between annual CT or MRI examination rates per 1000 population and the population share of each age group, expressed per 1000 population [19, 20]. Positive values indicate that examination rates increased in parallel with the corresponding age-group share, while negative values indicate an inverse association

### Population-level utilization and device-level productivity

Population-level utilization and examinations per scanner showed divergent cross-country patterns (Table 5). For CT, Canada and France recorded the highest device-level productivity (11,526.5 and 10,864.4 examinations per scanner, respectively), despite not having the highest population-level rates; Canada, in particular, achieved this with only moderate CT utilization. Conversely, Japan showed relatively high CT utilization but among the lowest examinations per scanner (2153.3 in 2014), a divergence mirrored for MRI (2172.4 per scanner). For MRI, France combined high population-level utilization with the highest device-level productivity (7863.2 examinations per scanner in 2023). These findings indicate that countries with lower scanner productivity may have greater scope to absorb future demand growth through operational improvements, while those already operating at high productivity will face greater workload pressure as demand rises.

**Table 5:** Population-level utilization and device-level productivity of CT and MRI scanners in selected countries, latest available year

Country	Year	CT exams (per 1000 persons)	CT examinations per scanner	MRI exams (per 1000 persons)	MRI examinations per scanner
Australia	2024	177.1	2369.20	54.8	3215.40
Canada	2022	164	11,526.50	56.9	5494.20
France	2023	228	10,864.40	150.8	7863.20
Germany	2023	168.5	4544.70	161.9	4587.10
Italy	2023	111.1	2678.80	82.8	2460.20
Japan	2014	221.5	2153.30	110	2172.40
Korea	2023	333.5	7358.50	90.3	2336.20
Spain	2023	150.8	6936.40	125.1	5730.30
USA	2021	254.5	5971.70	107.5	2831.10

CT and MRI examination rates are expressed as examinations per 1000 population. Examinations per scanner represent the annual number of CT or MRI examinations performed per scanner. The year refers to the most recent available observation in which both utilization rates and examinations-per-scanner data were available for each country [22]. For Japan, values refer to the single available OECD observation in 2014 [20]

## Discussion

### Principal findings

This study examined CT and MRI utilization trends across selected OECD countries, projected examination rates to 2050, and assessed their relationship with demographic ageing and scanner productivity. Three main findings emerged. First, CT and MRI utilization showed marked cross-country heterogeneity in both examinations per 1000 population and examinations per scanner, consistent with previous OECD reports [22] and recent international analyses [6]. Second, the share of adults aged 65 years and older is rising in all included countries, and CT and MRI examination rates were strongly and positively correlated with this demographic shift in most of them, coherent with population-based evidence of substantially higher imaging use among older adults [10]. Third, the operational implications of future demand growth differ across health systems depending not only on population-level utilization but also on how intensively installed scanner capacity is used.

### Demographic ageing and cross-country heterogeneity

Population ageing represents a predictable, long-term pressure on diagnostic imaging services, and its key planning advantage lies precisely in its gradual, anticipatable nature. Unlike sudden policy shifts or technological disruptions, demographic change gives health systems the opportunity to plan imaging capacity, workforce needs, and efficiency strategies proactively. However, the wide variation in CT and MRI examination rates across countries with similar ageing trajectories confirms that demographic structure alone does not explain observed utilization levels. System-level factors (clinical practice, technology diffusion, reimbursement models, and health system organization) play a substantial independent role [6, 23]. The divergence between population-level utilization and examinations per scanner further illustrates this point: as reported by Martella et al, examination volumes and device availability do not necessarily increase at the same pace, making the exam-to-scanner ratio a critical indicator of operational use [6]. Planning based solely on scanners per million population is therefore insufficient; a more informative approach should integrate population-level demand, installed capacity, examinations per scanner, and workforce availability [24].

### Capacity expansion and efficiency-oriented strategies

Physical capacity expansion is often the immediate response to rising imaging demand, and in settings where scanner productivity is already high, it may be necessary. However, adding scanners without addressing operational inefficiencies risks generating additional utilization without improving appropriateness, access, or sustainability. Efficiency-oriented strategies offer a complementary pathway, including reducing scanner idle time, optimizing scheduling, extending operating hours, standardizing protocols, reducing avoidable repeat examinations, and strengthening appropriateness criteria. Guidelines such as the American College of Radiology Appropriateness Criteria were developed to support referring physicians in selecting the most appropriate imaging examination [25]. Similarly, the Royal College of Radiologists’ *iRefer* guidelines provide an evidence-based framework for appropriate imaging referral and can be accessed through the *iRefer* clinical decision support system [26]. Evidence shows that guideline implementation and clinical decision support can meaningfully improve imaging-request appropriateness [27].

Technological innovation (faster acquisition protocols, reconstruction algorithms, workflow automation, and Artificial Intelligence-based decision-support tools) can further increase throughput without proportional infrastructure expansion. Sustainable planning does not require choosing between expansion and efficiency: the two approaches should be combined according to local context.

### Economic, environmental, and social sustainability

The implications of increasing CT and MRI demand extend beyond direct costs. Economically, both modalities require continuous investment in equipment, maintenance, infrastructure, and specialized staff, making clinical appropriateness and operational efficiency essential counterweights to volume growth. Environmentally, CT and MRI contribute meaningfully to hospital energy consumption; Heye et al demonstrated that relevant energy- and cost-saving opportunities exist particularly during non-productive operating states [28], and more recent reviews have confirmed that radiology departments can reduce energy use and carbon emissions through equipment management, optimized protocols, and workflow changes [29].

From a workforce perspective, rising examination volumes translate into additional pressure on radiologists, radiographers, and administrative staff at a time when demand is already increasing faster than workforce capacity in several settings [30]. Illustrative evidence from the United Kingdom is provided by the 2025 Royal College of Radiologists Clinical Radiology Workforce Census, which reported a 32% shortfall in clinical radiologists, projected to reach 40% by 2030, while demand for imaging was growing at more than twice the rate of workforce expansion [31]. These findings reinforce that imaging appropriateness is important not only for controlling expenditure and avoiding unnecessary examinations but also for reducing reporting backlogs, preserving limited specialist capacity for clinically indicated studies, supporting timely diagnosis, and protecting patient safety. Workforce capacity requires long planning horizons and cannot be expanded as readily as physical infrastructure; efficiency strategies can mitigate but not substitute for sustained investment in workforce development.

### Limitations

This analysis used country-level aggregate data and could not account for regional differences, indication-specific utilization, patient-level characteristics, or within-country disparities in access.

Although OECD Health Statistics provides a widely used basis for international comparisons, the underlying data are not fully homogeneous across countries. Differences in national reporting systems, coding practices, data completeness, and the inclusion of hospital, ambulatory, publicly funded, or privately funded examinations may systematically affect reported utilization and scanner-productivity estimates. Population estimates from the United Nations undergo consistency and quality assessments, but their accuracy also depends on the completeness and timeliness of national censuses, population registers, and vital statistics. In addition, Japanese utilization data were derived from an administrative claims database rather than from the longitudinal OECD series. Consequently, measurement errors or systematic differences in source coverage may propagate through the correlations and projections; therefore, absolute cross-country comparisons should be interpreted cautiously, with greater emphasis placed on broad trends and within-country trajectories.

Projections were based on linear extrapolation of historical examination rates and should be read as trend-based scenarios, not forecasts capable of incorporating future changes in clinical guidelines, technology, reimbursement policies, or disease prevalence. Correlations are descriptive and do not imply causality. Moreover, because both imaging utilization and population age structure changed over time, the high correlation coefficients may partly reflect shared temporal trends rather than a direct relationship between demographic ageing and imaging use. For Japan, longitudinal examination rates were derived from NDB Open Data, while scanner productivity data were limited to a single OECD observation from 2014; comparisons involving Japan should therefore be interpreted with caution.

## Conclusions

CT and MRI utilization increased across the selected OECD countries during the observed periods and, under the linear extrapolation scenarios applied in this study, may continue to rise to 2050. Demographic ageing was strongly associated with imaging utilization in most countries, consistent with the higher use of advanced imaging among older adults. However, these descriptive associations do not establish causality, and differences in examination rates and examinations per scanner suggest that future pressure on imaging services may also depend on health system organization, scanner productivity, and workforce capacity.

Sustainable imaging service planning may therefore benefit from considering demographic trends alongside appropriateness management, operational optimization, technological innovation, and workforce development. Capacity expansion may be necessary in some settings, but it should be guided by local productivity, demand trends, and sustainability goals rather than by examination growth alone.

## Data Availability

The data analyzed in this study are publicly available from the OECD Data Explorer, the National Database of Health Insurance Claims and Specific Health Checkups of Japan (NDB) Open Data, and the United Nations World Population Prospects 2024. The datasets used and analyzed during the current study are available from the corresponding author upon reasonable request.

## Abbrevations

CT: Computed tomography
MRI: Magnetic resonance imaging
NDB: National Database of Health Insurance Claims and Specific Health Checkups of Japan
OECD: Organization for Economic Co-operation and Development
PI: Prediction interval
